# THE UNIQUE IMPACT OF THE COVID PANDEMIC ON PREDOCTORAL GEROPSYCHOLOGY TRAINING: RECOMMENDATIONS FROM THE FIELD

**DOI:** 10.1093/geroni/igac059.302

**Published:** 2022-12-20

**Authors:** Candice Reel, Hannah Bashian, Julia Boyle, Mary Jacobs, Michelle Mlinac

**Affiliations:** The University of Alabama, Tuscaloosa, Alabama, United States; VA Boston HCS, Boston, Massachusetts, United States; New England GRECC, Boston, Massachusetts, United States; Tuscaloosa VA Medical Center, Tuscaloosa, Alabama, United States; VA Boston HCS, Boston, Massachusetts, United States

## Abstract

The COVID-19 pandemic impacted predoctoral psychology training at the graduate, practicum, and internship levels including a greater reliance on telehealth and evolving learning needs. However its unique impact on geropsychology training has not been explored. The Building Bridges workgroup for predoctoral training faculty levels, we describe opportunities and barriers to address evolving geropsychology training needs during the pandemic as determined through working group discussion. Negative impacts to training identified include: decreased opportunities for 1) face-to-face patient care and 2) telehealth care due to disparities in telehealth access and utilization in older adults. Other impacts on the geropsychology pipeline include declining opportunities to see older adults at practicum sites. Conversely, increased media attention to the impact of COVID on older adults’ physical and mental health may lead to graduate students’ having greater interest in geriatric mental health and reinforcing a geropsychology career. Recommendations for training programs to address the long-term ramifications of the pandemic will be offered.

